# Impact of Wechat-based prehospital data transmission on reperfusion times in ST-elevation myocardial infarction: a prospective observational study

**DOI:** 10.1016/j.clinsp.2026.101188

**Published:** 2026-09-30

**Authors:** Shangchen Li, Haibo Wu, Ying Wang, Yanqing Wang, Yuyang Xiao, Dan Yang, Dongxia Liu

**Affiliations:** a School of Graduate, Hebei Medical University, Hebei General Hospital, Shijiazhuang, Hebei Province, PR China; b Department of Cardiology, Hebei General hospital, Shijiazhuang, Hebei Province, PR China; c Department of Clinical Laboratory, Hebei General Hospital, Shijiazhuang, Hebei Province, PR China

**Keywords:** Acute ST-segment elevation myocardial infarction, Emergency medical services, Prehospital delay, Digital health, WeChat-based transmission

## Abstract

**Objectives:**

Early diagnosis of acute ST-segment Elevation Myocardial Infarction (STEMI) is important for the treatment of patients. A Class I evidence demonstrates that every 30-minute delay in PCI is linked to a 7.5% relative increase in 1-year mortality^1^ “WeChat” is a convenient communication tool that enables the transmission of patient information ‒ including basic details, condition assessments, and relevant laboratory results ‒ to the receiving hospital via WeChat-based transmission. Simultaneously, patients are rapidly transported to the hospital by ambulance for treatment. Thus, the hospital can receive patient information before their arrival, thereby significantly reducing diagnosis and treatment time. This integrated model is collectively referred to as WeChat-based transmission. Therefore, the early activation of WeChat-based emergency medical services transmission plays a critical role in improving patient prognosis. The present study sought to investigate the use of WeChat-based transmission in patients with acute STEMI.

**Methods:**

409 eligible STEMI patients (May 2022–November 2024) were grouped by WeChat-based transmission use: 96 in the observation group and 313 in the control group. Baseline data, medical history, and key times (S-to-FMC, d-to-B, TIT) were compared. Factors influencing WeChat-based transmission use were analyzed via SPSS 22.0 (p < 0.05 significant).

**Results:**

96 (23.4%) patients in the WeChat-based transmission group and 313 (76.6%) patients in the N-WeChat group were enrolled. S‑to‑FMC time was associated with lower median values in the WeChat‑based transmission group (median 75 vs. 120 minutes, p < 0.05); similar patterns were seen for D‑to‑B time (50 vs. 67 minutes, p < 0.05) and total ischemia time (121.5 vs. 150.0 minutes, p < 0.05). Shorter time to Acute Myocardial Infarction (AMI) diagnosis, as well as shorter D‑to‑B and TIT times, were observed among patients in the WeChat‑based transmission group relative to patients in the non‑WeChat‑based transmission group.

**Conclusion:**

WeChat-based transmission was associated with shorter S-to-FMC, d-to-B, and TIT times during patient transfer for PCI. However, it is not fully used for transporting chest pain patients in and around Shijiazhuang City. Therefore, strengthening WeChat-based transmission function in public education of pre-hospital rescue remains the focus of our future work.

## Highlights

• Timely treatment is crucial for acute myocardial infarction patients.
• WeChat facilitates timely transfer of patient data between hospitals.
• Early data transmission shortens treatment time after hospital arrival.
• WeChat-based data transfer shows significant promise in the medical field.
• Findings support the potential value of hospital collaboration in regional STEMI care systems.

## Introduction

### Epidemiological research on AMI

Acute Myocardial Infarction (AMI) is the most severe manifestation of Coronary Heart Disease (CHD). It is associated with several serious complications, and its incidence is increasing among younger people. The primary goal in the emergency treatment of patients with ST-segment Elevation Myocardial Infarction (STEMI) is to initiate early reperfusion therapy, either through thrombolysis or Percutaneous Coronary Intervention (PCI). Early reperfusion is essential to reduce infarct size, maintain left ventricular function, and improve both short-term and long-term outcomes in STEMI patients^1^, ^2^, ^3^ However, delays in reperfusion therapy remain a global challenge. Evidence has shown that reperfusion treatment within the first hour of symptom onset is most effective in STEMI patients. However, treatment delayed beyond 3-hours significantly diminishes outcomes^4^

The time from symptom onset to First Medical Contact (S-to-FMC) and Door-to-Balloon (D-to-B) largely determine the time of reperfusion treatment for patients with chest pain. Notably, the establishment of chest pain centers was associated with significantly shorter d-to-B times, owing to well-coordinated care provided by in-hospital medical teams. Accordingly, shortening pre-hospital delay was identified as the key measure to reduce TIT.

Through improvements in WeChat-based transmission, and the establishment of regional collaborative treatment have significantly reduced reperfusion treatment times in China. Nonetheless, further improvements are still needed. There are no chest pain data in Shijiazhuang and surroundings were reported, and the effects of regional collaborative treatment systems in chest pain rescue is due to be confirmed.

China has a vast territory with unevenly distributed medical resources. Patients in rural or remote areas often require long-distance transfer to higher-level hospitals because local hospitals lack PCI capability. The key to regional collaborative care is to identify the nearest PCI center rapidly and realize efficient coordination based on the principle of “information arriving before the patient”.

A study covering multiple chest pain centers in China demonstrated that after implementing a regional collaborative care network based on remote Electrocardiogram (ECG) transmission, the median Door-to-Balloon (D-to-B) time was significantly reduced from over 90-minutes to less than 70-minutes, with the best-performing centers consistently achieving d-to-B times below 50-minutes. Another study analyzing a large regional care system in the United States, which integrated 21 non-PCI hospitals and 4 PCI centers through a cloud-based platform, reported a 20% reduction in d-to-B time for referred patients and a downward trend in mortality, providing an international reference for establishing similar systems^5^, ^6^, ^7^

### The concept of wechat-based transmission

WeChat is a national-level multifunctional social application developed by a Chinese enterprise. It deeply integrates communication, payment, lifestyle services, and a mini-program ecosystem, serving as an indispensable app for Chinese users in daily social interactions, consumption, and administrative affairs. Users can send text/voice messages, make video calls, and more through it, greatly facilitating mutual communication among people.

To initiate a group chat, simply invite two or more members. Within the group chat, it enables medical professionals to transmit patient information ‒ including basic details, condition assessments, and relevant laboratory results ‒ to the receiving hospital via WeChat-based transmission. Simultaneously, the patient is rapidly transported to the hospital by ambulance for treatment. This allows the receiving physicians to access the patient’s information before their arrival, providing sufficient time to evaluate the condition. As a result, it significantly reduces the time required for diagnosis and treatment after the patient arrives at the hospital, enabling faster access to interventional therapy. WeChat has become an essential digital hub in the daily lives of Chinese people. This model is collectively referred to as WeChat-based transmission.

The present study analyzed the application of the WeChat-based transmission model among STEMI patients undergoing interventional therapy, as well as its impact on symptom onset-to-treatment times and subsequent PCI. This study sought to identify existing challenges in the use of WeChat-based transmission in chest pain management.

## Methods

### Subjects and questionnaires

#### Subjects

A total of 421 patients with acute STEMI from Shijiazhuang City, Hebei Province, China and surrounding areas (within 60-minutes of transport time) undergoing primary PCI at the Cardiac Care Unit (CCU) of Hebei General hospital from May 2022 to November 2024 were recruited. A unified questionnaire was used by trained and qualified investigators to prospectively collect data in real‑time from patients residing in Shijiazhuang City, Hebei Province during their clinical care. The inclusion criteria were as follows: 1) Typical symptoms of ischemic chest pain; 2) STEMI confirmed by the hospital’s initial Electrocardiogram (ECG); 3) An indication for PCI; 4) Informed consent from both the patients and their family members; and 5) Acute chest pain with onset within 24-hours, treated with PCI. The exclusion criteria were as follows: 1) Contraindication of PCI; 2) Receipt of thrombolytic therapy; 3) Incomplete records of crucial time points; 4) Complications, such as malignant tumors, immune system diseases, severe hepatic or renal insufficiency, infectious diseases, etc.; 5) Unconsciousness, receipt of endotracheal intubation, or death during hospitalization and 6) End‑stage illness, in‑hospital onset, or declined participation in the study. A total of 409 patients met the inclusion criteria.

Taking the Door‑to‑Balloon (D‑to‑B) time as the primary outcome indicator, sample‑size calculation was performed using PASS 22.0 software for proportion comparison. Based on previously published real‑world pre‑hospital ECG‑transmission studies among Chinese STEMI populations, we anticipated a median reduction of approximately 15–20 min in D‑to‑B time for patients receiving pre‑hospital digital information transmission, which served as our expected effect size for power calculation. The assumed 20%‒25% utilization rate of WeChat‑based transmission was derived from observational data of regional pre‑hospital tele‑ECG programs in similar Chinese chest‑pain center cohorts. Statistical parameters were set as α = 0.05 (two‑tailed) and β = 0.2 (corresponding to a statistical power of 80%). The minimum required sample size was calculated as 380 cases. The final enrolled sample of 409 patients satisfied the statistical requirement and provided an adequate buffer to account for potential data loss or deviations from the presupposed effect size and utilization rate.

#### Questionnaires

Trained and qualified investigators used a uniform questionnaire to collect patient data, including demographics (name, sex, age, marital status, education level, work status, contact information, physical examination findings, and biochemical indicators), medical history, family history, symptom characteristics, the mode of transportation from symptom onset to the First Medical Institution(FMC), and the mode of transportation to the PCI institution, and whether the information was transmitted to the hospital via WeChat-based transmission. Informed consent was obtained from all participants.

### WeChat-based transmission

For primary PCI patients transported under the WeChat-based transmission protocol at first medical contact, and the mode of transportation to the PCI center, as well as whether information was transmitted to the hospital via WeChat-based transmission. The patient is then administered dual antiplatelet therapy within 15 minutes, along with intravenous heparin at a dose of 50 IU/kg. Patient information ‒ such as age, sex, medical history, time of symptom onset, symptoms, and laboratory results ‒ is shared in the WeChat-based transmission. Upon receiving this information, the operator activates the catheterization laboratory while the patient is en route to the hospital. As a result, most patients can be transferred directly to the cath lab, bypassing the emergency department and CCU.

Decision-making for emergency department bypass and direct transfer to the catheterization laboratory followed local chest-pain-center criteria: patients with STEMI confirmed by pre-hospital ECG, without severe hemodynamic instability or life-threatening arrhythmia, were eligible for ED bypass. Patients with unstable vital signs would be diverted to the emergency department for initial resuscitation. Real-time communication within the WeChat group allowed the interventional cardiologist to screen risks in advance; emergency resuscitation resources were always on standby as a safety backup.

For transferred PCI cases, clinicians at the regional collaborative center transmit patient information to the dedicated group via WeChat-based transmission, where treatment suggestions can also be provided. The catheterization laboratory is activated immediately, and the emergency department and CCU are similarly bypassed.

This WeChat‑based framework enables ongoing real‑time sharing of patient information and timely preparation of the catheterization laboratory. Physicians performing PCI were highly experienced in emergency interventional treatment, with 95% of procedures conducted via the radial artery to minimize complications and enhance patient comfort.

For inter-hospital communication, a dedicated WeChat-based transmission group was established. Patient Electrocardiograms (ECGs), along with other relevant laboratory results and estimated arrival times, were transmitted through these groups. To ensure data security and patient privacy, no personally identifiable information ‒ such as patient names, ID numbers, or home addresses ‒ was shared in the groups. Furthermore, access to these groups was strictly controlled: membership was restricted to authorized personnel only, and all prospective members underwent a verification process by designated administrators prior to joining. These measures provided comprehensive protection for both the transmitted data and patient information.

All pre-hospital emergency personnel participating in the WeChat-based transmission workflow received unified on-the-job training covering required content for pre-hospital information submission. A standardized checklist was adopted for information upload, including patient age, key medical history, symptom onset time, vital signs, 12-lead ECG findings, and estimated hospital arrival time, to guarantee complete and consistent information delivered within the WeChat group.

### Diagnostic criteria

#### Diagnostic criteria for AMI

According to the 2019 guideline for the diagnosis and treatment of acute STEMI in China, the diagnostic criteria of AMI included clinical evidence of myocardial injury and ischemia, characterized by 1) Myocardial ischemia symptoms; 2) New ischemic changes on ECG; 3) The presence of pathological Q waves; 4) Imaging evidence of newly viable myocardium loss or abnormal ventricular wall motion of ischemic origin; 5) Identification of coronary thrombosis through angiography or autopsy.

#### S-to-FMC time

S-to-FMC time was defined as the time from symptom onset to the first medical contact with a physician, paramedic, or other emergency responders capable of providing ECG and initial treatment.

Symptom onset time was recorded based on patients’ or family caregivers’ self-report. Paramedics and emergency physicians were trained to conduct standardized inquiry about symptom onset, including the exact time point when chest pain or related discomfort first began. Nevertheless, retrospective self-reported onset time is subject to recall bias in emergency clinical settings, which is acknowledged as one limitation of this study.

#### **D**-to-B time

D-to-B time was defined as the time from hospital door entry to the first balloon expansion.

#### TIT time

TIT time was defined as the time from symptom onset to balloon expansion.

### Statistical analysis

Statistical analysis was performed using SPSS 22.0. Tested by Shapiro-Wilk to confirm whether the distribution is normal. Normally distributed continuous variables were presented as the mean ± standard deviation and compared using a Student’s *t*-test. Non-normally distributed continuous variables were expressed as the median Interquartile Range (IQR: 25th, 75th percentile) and compared using a rank-sum test (Mann-Whitney *U test*). A Chi-Square (χ^2^) test was employed for categorical data. Univariate and multivariate logistic regression analyses were used to evaluate factors affecting the application of WeChat-based transmission; p < 0.05 was considered statistically significant.

## Results

### Comparison of patient characteristics between wechat-based transmission and N-WeChat groups

A total of 409 participants were included in the final analysis, with a mean age of 60.2 ± 11.9 years. The symptom onset to first medical contact time (S-to-FMC), defined as the time from symptom onset to contact with physicians, paramedics and other emergency responders capable of performing ECG and delivering initial treatment, was a median of 115 (58, 223) minutes. According to whether patient information was shared via WeChat before ambulance transportation, all patients were divided into two groups. In the WeChat‑based transmission group, prehospital data was shared in real time via WeChat during ambulance transport, while the non-WeChat‑based transmission group received routine ambulance transport without digital data sharing. The WeChat‑based transmission group consisted of 96 patients (23.4%), and the non-WeChat‑based transmission group included 313 patients (76.6%).

No significant differences were found between the WeChat-based transmission and N-WeChat groups regarding age, gender, body mass index, or biochemical indexes. The proportion of patients with a high school education or above was significantly higher in the WeChat-based transmission group than in the N-WeChat (p < 0.05). No difference was found between the two groups in terms of medical history and family history, except for a higher proportion of patients with a history of CHD in the WeChat-based transmission than in the N-WeChat group (p < 0.05). Shorter S‑to‑FMC and D‑to‑B times were observed in the WeChat‑based transmission group relative to the N‑WeChat group. (p < 0.05) (Table 1).

**Table 1:** Comparison of baseline information between WeChat-based transmission and N-WeChat groups.

Variable	WeChat-based transmission (n = 96)	N-WeChat-based transmission (n = 313)	p-values
Age (years)^a^	62.13 (±1.42)	59.66 (±0.82)	0.139
Male, n (%)	69 (71.4)	249 (79.4)	0.134
BMI^a^	25.58 (±3.47)	25.99 (±3.86)	0.450
UA^a^	356.06 (±76.64)	497.33 (±96.04)	0.324
TG^b^	1.54 (1.17, 2.09)	1.53 (0.98, 2.12)	0.721
TC^b^	4.60 (4.05, 5.24)	4.53 (3.93, 5.22)	0.809
LDL-c^a^	1.07 (±0.26)	1.01 (±0.21)	0.086
HDL-c^a^	3.10 (±0.98)	3.06 (±0.81)	0.753
Glu^b^	6.25 (4.71, 7.77)	5.60 (4.92, 7.52)	0.646
S-to-FMC^b^	75.0 (30, 205)	120 (60, 138)	0.023
D-to-B^b^	50 (39, 63)	67 (57, 78)	0.045
TIT^b^	121.5 (104.5, 296)	150.0 (115.0, 333.0)	0.028
Education level (high school education or above), n (%)	49 (51.0)	109 (34.9)	0.049
Hypertension, n (%)	52 (54.2)	134 (42.7)	0.092
Diabetes, n (%)	23 (23.9)	75 (23.9)	1.000
Cerebrovascular disease, n (%)	18 (18.8)	64 (20.5)	0.792
Family history of coronary heart disease, n (%)	44 (45.8)	126 (40.4)	0.399
angina pectoris appeared 48 h before onset, n (%)	54 (56.3)	139 (44.5)	0.094
Typical symptoms, n (%)	62 (64.6)	201 (64.2)	0.658
Treatment history of coronary heart disease, n (%)	10 (10.4)	8 (2.6)	0.015
Onset at night, n (%)	49 (51.4)	171 (54.6)	0.673
Patient education, n (%)	23 (24.3)	56 (17.9)	0.298

BMI, Body Mass Index; UA, Uric Acid; TG, Triglyceride; TC, Total Cholesterol; LDL-c, Low-Density Lipoprotein cholesterol; HDL-c, High-Density Lipoprotein cholesterol; Glu, Glucose.

a Variables with normal distribution: presented as mean ± Standard Deviation (SD), compared by *t*-test.

b Variables with non-normal distribution: presented as median (25th, 75th percentiles), compared by rank-sum test. Note: The variable “Patient education” in Table 1 is identical in definition to “Patient education” presented in Table 2.

### Logistic regression analysis

#### Univariate analysis

Univariate analysis was conducted to identify factors affecting the choice of WeChat-based transmission at first medical contact (Table 2). It was found that a history of CHD, gender, education level (high school or above), the elderly (> 65-years-old), participation in patient education, perception of symptom severity within 1-hour of onset, suspected heart disease, angina pectoris occurring 48-hours before onset, symptom onset at home, awareness of myocardial infarction symptoms, symptoms onset, and history of CHD treatment were associated with WeChat-based transmission application.

**Table 2:** Univariate analysis of factors affecting patient’s choice of WeChat-based transmission.

	WeChat-based transmission (n = 96)	N-WeChat-based transmission (n = 313)	χ^2^ value	p-value
Male, n (%)	69 (71.4)	249 (79.4)	2.468	0.134
Education (high school or above), n (%)	49 (51.4)	109 (34.9)	4.226	0.049
Elderly (over 65 years old), n (%)	37 (38.6)	105 (33.5)	0.283	0.347
Family history of coronary heart disease, n (%)	31 (44.3)	120 (38.4)	0.115	0.418
Take seriously within 1h of onset, n (%)	26 (37.1)	149 (47.6)	2.576	0.125
Suspected heart disease, n (%)	37 (38.6)	131 (41.9)	0.292	0.346
Patient education, n (%)	23 (24.2)	56 (17.9)	1.322	0.164
Angina pectoris appeared 48-hours before onset	52 (54.3)	132 (42.3)	3.070	0.094
Onset at night, n (%)	49 (51.4)	125 (54.6)	0.271	0.351
Onset in home, n (%)	75 (78.6)	204 (65.1)	4.757	0.031
Treatment history of coronary heart disease, n (%)	10 (10.4)	8 (2.6)	9.972	0.015

#### Multivariate logistic regression analysis

Subsequently, multivariate logistic regression analysis was conducted on factors that showed significant differences in univariate analysis (p < 0.1). The study found that education level, symptom onset at home, and history of CHD treatment were independent factors significantly associated with WeChat-based transmission application after symptom onset (Table 3).

**Table 3:** Multivariate logistic regression analysis of factors affecting patient’s choice of WeChat-based transmission.

	Regression coefficient	Wald values	OR	95% CI	p-value
Education (high school or above)	0.581	4.620	1.771	1.007, 3.114	0.032
Onset in home	0.827	5.373	2.286	1.136, 4.600	0.020
Treatment history of coronary heart disease	−1.402	5.594	0.246	0.077, 0.787	0.018

### Relationship between wechat-based transmission and S-to-FMC, **D**-to-B, and TIT times

To further investigate the efficacy of WeChat‑based transmission and account for potential confounding effects from inter‑hospital transfer distances on outcome assessment, we performed subgroup analyses restricted to patients with well‑defined transport pathways: 141 patients who were initially transported by ambulance directly to our hospital, and 251 patients who were transferred by ambulance from other hospitals to our institution, summing to 392 patients. The remaining 17 of the total 409 enrolled patients were excluded from this stratified subgroup analysis due to ambiguous transport routes, including self‑presentation by private vehicle without ambulance emergency service involvement and incomplete documentation of transfer pathways. Each of the 392 analyzable patients was subsequently stratified into two groups based on whether WeChat‑based transmission was implemented during transport (Fig. 1).

**Fig. 1:**
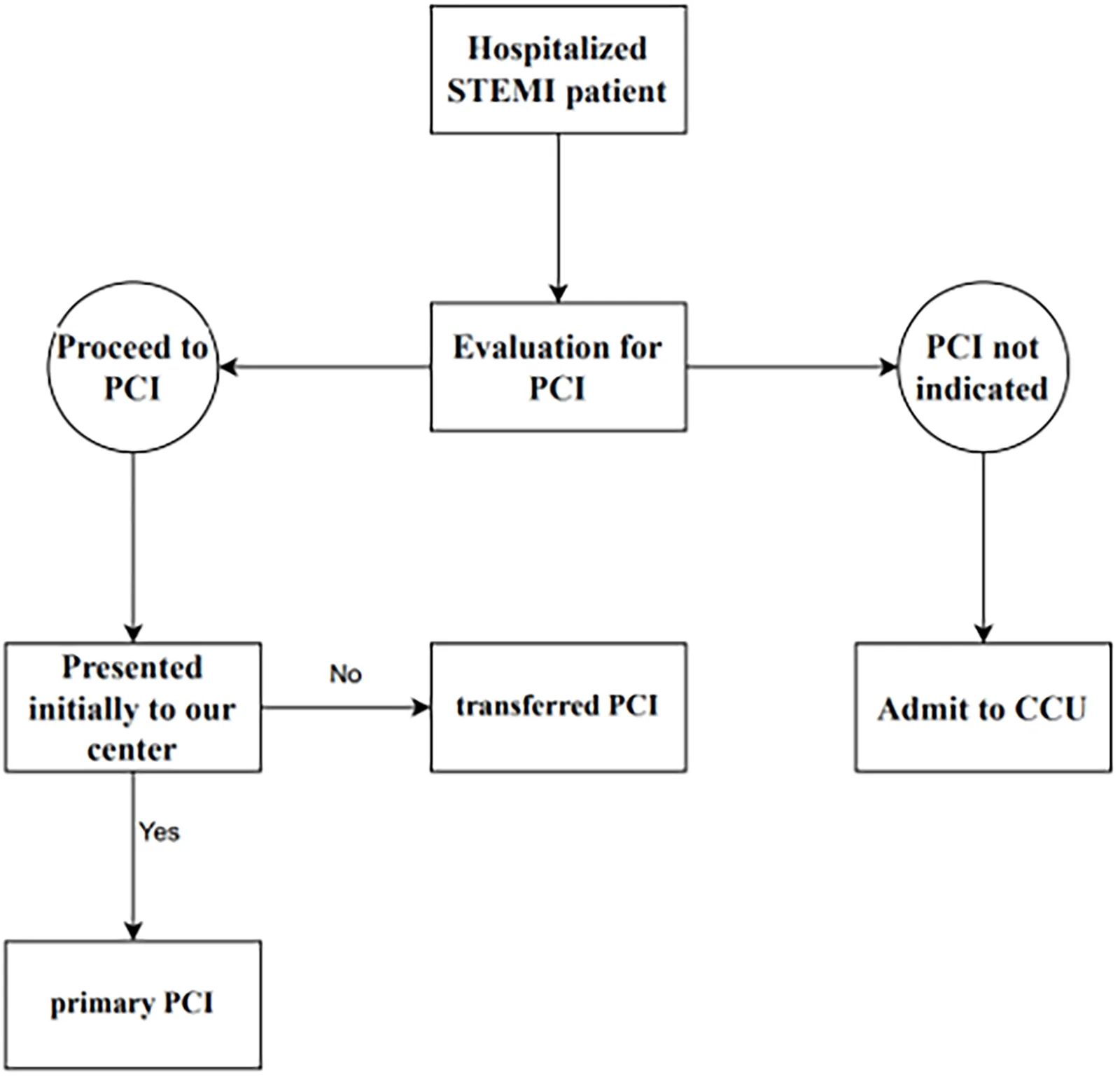
Management pathway: direct PCI or transfer for PCI.

#### Effects of wechat-based transmission on S-to-FMC, **D**-to-B, TIT times in patients with primary PCI

A subgroup analysis was performed on 141 patients receiving direct PCI at our hospital as the initial presentation site to assess the impact of WeChat-based transmission on treatment timelines. Primary PCI is defined as the PCI procedure performed at our hospital on patients who presented here directly, as opposed to those transferred from other facilities (Transfer PCI). Among them, 52 patients (36.9%) were in the WeChat-based transmission group, with a median S-to-FMC time of 75 (30‒205) minutes, d-to-B time of 50 (39‒63) minutes, and TIT time of 121.5 (104.5‒296) minutes. The 89 patients (63.1%) in the N-WeChat group had an S-to-FMC time of 120 (60‒138) minutes, d-to-B time of 67 (57‒78) minutes, and TIT time of 150.0 (115.0‒333.0) minutes (p < 0.05), with significant statistical differences.

The rank-sum test results showed significant differences between the two groups regarding S-to-FMC, d-to-B, and TIT times (p < 0.05) (Fig. 2). The scatter plot of the relationship among these time intervals showed that S-to-FMC time was linearly correlated with TIT time (*γ* = 0.588) (Fig. 3).

**Fig. 2:**
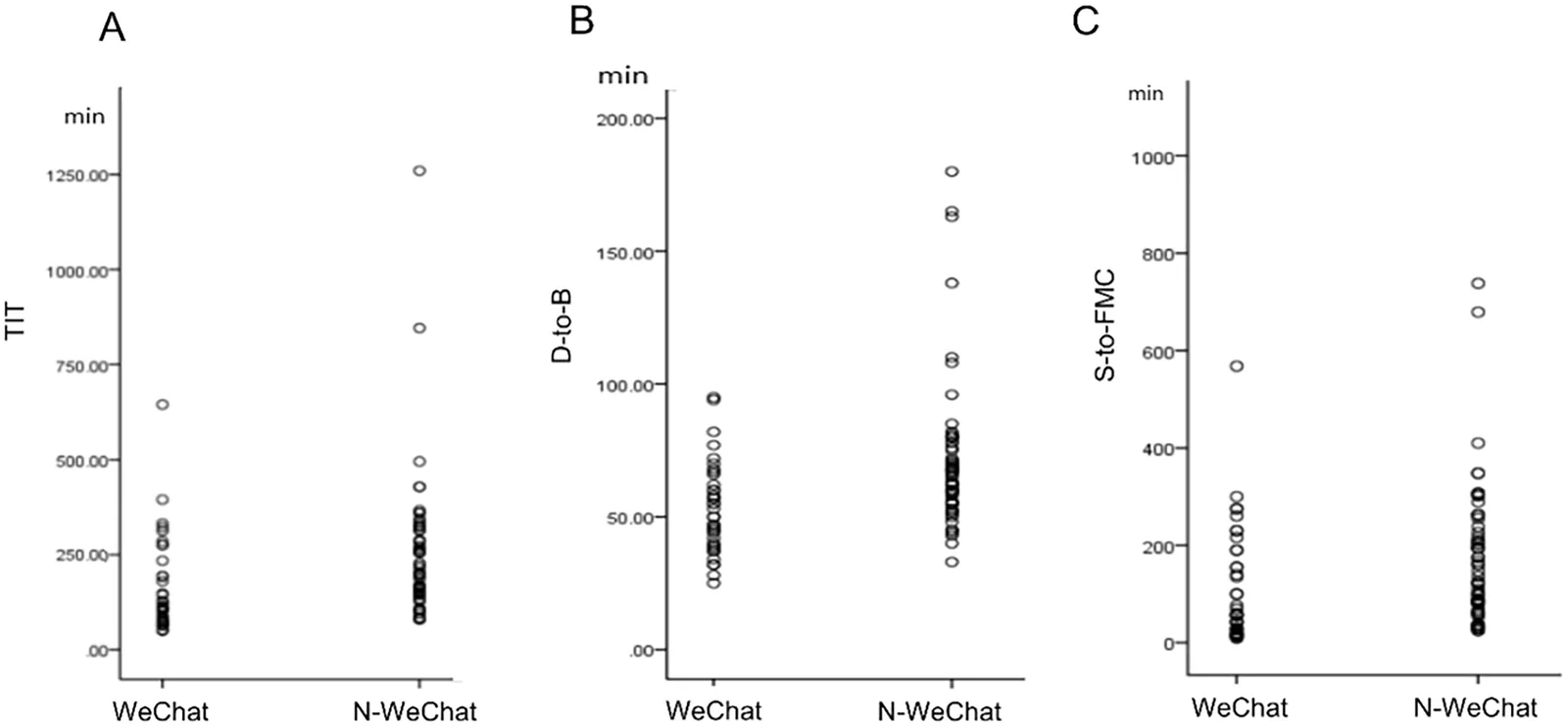
Effects of WeChat-based transmission on S-to-FMC, d-to-B, TIT in patients with primary PCI.

**Fig. 3:**
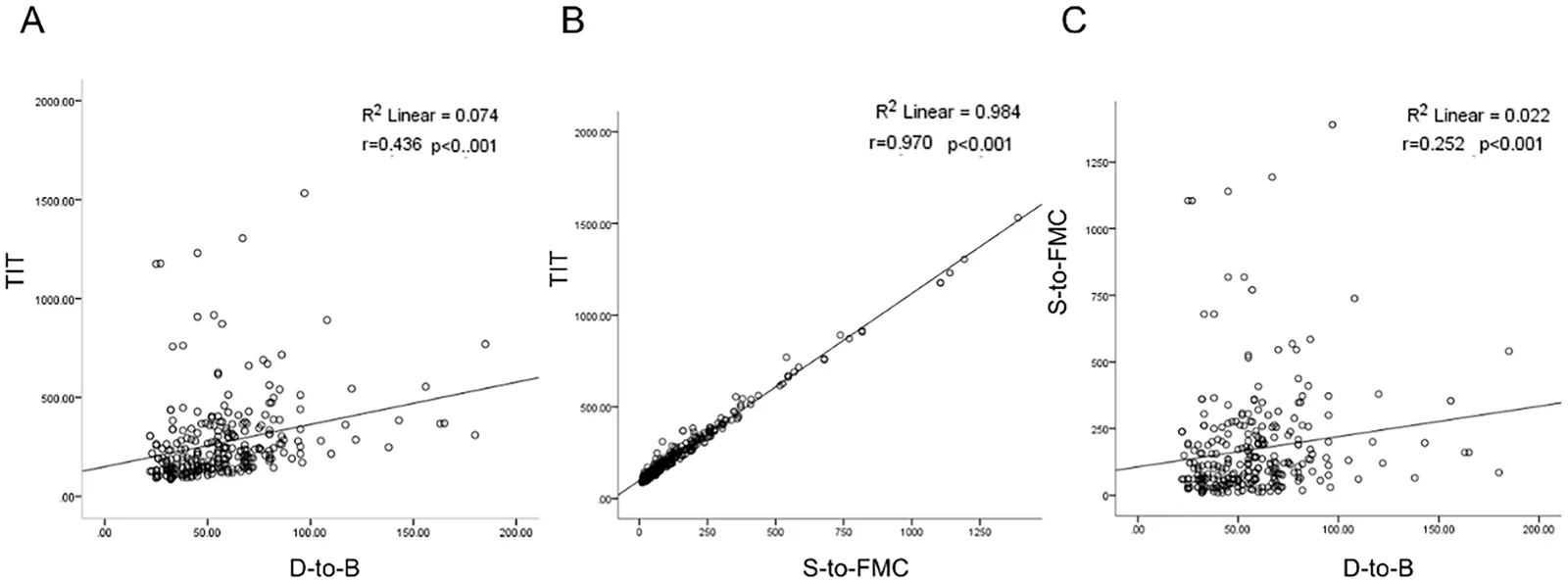
Scatter diagram and linear regression line of the time node relations of S-to-FMC, d-to-B and TIT.

#### Influence of wechat-based transmission on patients transferred for PCI

To investigate the impact of WeChat-based transmission on patients whose first medical contact was at a non-PCI hospital and required transfer to our facility for PCI, we conducted a further analysis of 251 transferred PCI patients. Patients transferred with WeChat-based transmission were defined as the WeChat-based transmission group. (WeChat-T, n = 170, 67.7%), while those without were assigned to the WeChat-NT group (WeChat-NT, n = 81, 32.3%). A total of 131 (76.8%) patients in the WeChat-based transmission group were directly transferred by WeChat-based transmission to the catheterization laboratory through the regional collaborative treatment system. The remaining patients needed further evaluation due to complex conditions and were transferred to our hospital’s emergency department or CCU, followed by primary PCI.

All patients in the WeChat-NT group were first taken to the emergency department to assess their condition, obtain informed consent, and perform primary PCI. The d-to-B time for the WeChat-based transmission and WeChat-NT groups were 44 (32‒58) and 65 (52‒79) minutes, respectively. The TIT differed significantly between the two groups: 194 (137‒296) minutes in the WeChat‑based transmission group versus 254 (153‒385) minutes in the WeChat‑NT group (p < 0.05). Shorter D‑to‑B and TIT times were observed in the WeChat‑based transmission group relative to the WeChat‑NT group. Additionally, Pearson Chi-Square test results revealed a statistically significant difference between the two groups in the proportion of patients who went directly to the catheter room (p < 0.01) (Table 4).

**Table 4:** Subanalysis on transfered PCI patients by WeChat-based transmission.

	WeChat-T (n = 170)	WeChat-NT (n = 81)	p-values
D-to-B (min**)**	44 (32, 58)	65 (52, 79)	<0.001
TIT (min**)**	194 (137, 296)	254 (153, 385)	0.039
Go straight to cath lab, n (%)	131 (76.8%)	0 (0.0%)	<0.001

Note: The reason for 0 cases (0.0%) of “going straight to the cath lab” in the WeChat‑NT group was that patients without WeChat‑based transmission could not be evaluated in advance. They required initial assessment and examinations in the emergency department before further management and thus could not be directly admitted to the cath lab for PCI. This was not a chance occurrence in the present sample. WeChat‑T, WeChat‑based Transmission group; WeChat‑NT, non‑WeChat‑based transmission group.

## Discussion

In summary, the WeChat-based transmission system serves as an “information expressway” for multi-hospital collaborative care, establishing a more efficient regional cooperative treatment system.

The observed value of the WeChat-based transmission model in this study stems from two interrelated mechanisms that transform traditional sequential care into parallel processing. First, it enables real-time data transmission and prospective decision-making. Upon first medical contact, ambulance personnel can immediately transmit critical information ‒ including ECG, vital signs, and medical history ‒ to the receiving hospital's dedicated WeChat-based transmission group. This allows the interventional team to conduct remote diagnosis, assess primary Percutaneous Coronary Intervention (PCI) indications, and activate the catheterization laboratory while the patient is still in transit, embodying the “information before patient” principle. Second, it facilitates direct catheterization laboratory access. With advanced notification and preparation, eligible patients can be transported directly to the catheterization laboratory, bypassing both the emergency department and coronary care unit. This eliminates redundant triage and evaluation steps, substantially reducing in-hospital delays. These mechanisms collectively were associated with reductions in both the prehospital and in-hospital components of treatment delay^8^

The WeChat‑based transmission model was associated with improved inter‑hospital physician collaboration; it may help avoid treatment delays stemming from inadequate capabilities at the referring hospital or prolonged assessments, and was associated with shorter total ischemia time^9^^,^^10^ This study found that the utilization of this model is closely related to the first-contact physician's understanding of its functions and chest pain management protocols. Therefore, increasing the utilization rate of WeChat-based transmission during post-symptom transfer is a key measure for optimizing treatment processes in patients with acute myocardial infarction.

Numerous studies have demonstrated that the implementation of such systems is associated with earlier initiation of pharmacotherapy, faster activation of the catheterization laboratory, earlier acquisition of informed consent for intervention, and shorter Symptom-to-Balloon (S-to-B) times^11^, ^12^, ^13^ Further comparative analysis revealed that the WeChat-based transmission group was associated with significantly shorter Symptom-to-First Medical Contact (S-to-FMC) and Door-to-Balloon (D-to-B) times compared with the non-WeChat group, suggesting an association between WeChat-based transmission and optimization of these critical intervals.

Multiple studies have indicated that, beyond clinical variables, sociodemographic characteristics such as age, gender, patient education level, and family support are also associated with whether patients call an ambulance and utilize WeChat-based transmission after symptom onset^14^^,^^15^ The present study found that patient education level (high school or above), onset at home, and a history of coronary heart disease treatment were independently associated with the use of WeChat-based transmission. These findings highlight important socioeconomic disparities and health literacy gaps that may influence access to digital emergency care. Higher educational attainment generally correlates with better ability to obtain and understand health information, greater familiarity with digital platforms, and higher health literacy, all of which facilitate the use of WeChat-based transmission systems. Notably, these differences may contribute to a digital divide in acute STEMI care: if such interventions are preferentially used by more educated and socially advantaged patients, they could potentially widen existing disparities in timely reperfusion unless targeted strategies are implemented to ensure equitable access for all patient groups. Meanwhile, the association with home onset may reflect two key issues: on the one hand, the home setting offers more stable conditions for seeking emergency care, particularly for elderly or less mobile patients; on the other hand, it also underscores gaps in emergency recognition and response systems in workplaces and other public areas^16^, ^17^, ^18^

Specifically, patients with an educational level above high school tend to place greater emphasis on their health, possess a more comprehensive understanding of acute myocardial infarction, and demonstrate greater familiarity with operating WeChat-based transmission functions. Data revealed that the proportion of highly educated individuals exposed to STEMI emergency knowledge through channels such as hospital public accounts and community health lectures was significantly higher than that of their less-educated counterparts (approximately 62% vs. 31%). This disparity in access to health information was correlated with the gradient distribution in WeChat-based transmission utilization rates. Notably, differences in health literacy are reflected not only at the knowledge level but also in the ability to translate knowledge into timely help-seeking behaviors ‒individuals with higher health literacy are better at recognizing the urgency of acute symptoms and are more trusting and adept at utilizing digital medical coordination services^19^, ^20^, ^21^, ^22^

Promoting the WeChat-based transmission regional collaborative care system requires addressing the “digital divide” in health literacy^22^ Future public health strategies should adopt a multidimensional approach: implementing targeted community education for populations with lower emergency knowledge, optimizing the WeChat-based transmission interface for elderly-friendly use, and exploring integration of digital emergency training into chronic disease management. These measures may help ensure that diverse socioeconomic groups can benefit from this technological innovation^24^, ^25^, ^26^, ^27^

Our findings align with and extend the growing body of evidence supporting digital health interventions in STEMI care. Internationally, studies have demonstrated that telemedicine systems, cloud-based platforms, and regional networks are associated with reduced reperfusion times^11^, ^12^, ^13^^,^^23^ The innovation of our study lies in leveraging WeChat ‒ a ubiquitous social media platform in China ‒ as a low-cost, highly accessible tool to explore achieving similar benefits. While dedicated telemedicine systems require substantial infrastructure investment, our approach utilizes existing digital literacy and communication patterns, offering a potential model for resource-variable settings. The observed time reductions (particularly in d-to-B time) are comparable to those reported in systems using more specialized technology,^23^ suggesting that platform selection may be less critical than the effective integration of digital communication into standardized clinical pathways.

Of note, multivariate logistic regression showed that patients with a history of prior coronary heart disease treatment were less likely to receive WeChat-based prehospital transfer. This may be explained by their long-term regular follow-up in cardiology clinics and established fixed medical-seeking pathways, which differ from those of patients with newly-onset STEMI. This phenomenon may reflect well-established doctor-patient relationships from routine outpatient follow-up. Such patients tend to follow their accustomed help-seeking routines when symptoms occur, rather than adopting newly-implemented pre-hospital digital transmission workflows.

Looking ahead, the WeChat-based transmission model also holds room for further optimization in real-world practice. Beyond its current function as an information channel, it holds potential to evolve into an intelligent regional rescue platform integrated with AI, IoT, and big data. Future developments may include embedding AI diagnostic modules to analyze ECGs within seconds, reducing reliance on manual interpretation. Integration with electronic health records could enable automatic patient data retrieval, improving accuracy and efficiency. Ultimately, WeChat-based transmission could transition from a communication tool into an intelligent coordination system for AMI care ‒ enabling smart diagnosis, real-time collaboration, and data-driven decision-making. This shift toward proactive, networked, and intelligent management promises to establish a more efficient, accessible regional treatment system, helping preserve myocardium and save lives.

### Limitations

We acknowledge several limitations of this study.

First, in terms of study design. This was a single-center observational study with a moderate sample size, which may introduce selection bias. Furthermore, the use of WeChat-based transmission was non-randomized, and baseline differences between groups may have independently influenced treatment intervals. Although we adjusted for measured confounders, residual confounding from unmeasured factors such as health literacy and socioeconomic status could not be completely ruled out.

Second, in terms of outcome measures. We did not perform subgroup analyses by urban-rural residence, which limited exploration of geographic and health system-related effects. In addition, our study focused on process indicators including time delays rather than hard clinical endpoints such as major adverse cardiovascular events or mortality. Although shorter ischemic time is closely associated with improved prognosis, clinical outcomes associated with WeChat‑based transmission remain to be definitively established.

Third, in terms of generalizability. Due to potential differences in digital infrastructure and population digital literacy, the application of this digital intervention may be influenced by a digital divide. Therefore, caution is warranted when generalizing these results to primary care settings or regions with limited resources. Future multicenter, Prospective randomized studies are needed to validate the robustness and generalizability of our findings.

Furthermore, symptom-onset time relied on self-report from patients or family caregivers. Despite standardized training for paramedics and emergency physicians to query the exact time of initial chest-pain-related discomfort, retrospective self-report in emergency conditions is subject to recall bias.

## Implications and conclusion

Despite these limitations, our study provides evidence supporting the integration of WeChat-based transmission (WeChat-T) into regional STEMI care systems. The implications are threefold:

For clinical practice: The protocol should be standardized and incorporated into chest pain center accreditation and quality control systems. Particular attention should be paid to training first-contact physicians at non-PCI-capable hospitals, as their engagement appears closely related to utilization rates.

For public health strategy: To promote equitable distribution of benefits, technology implementation must be accompanied by targeted health literacy initiatives. This includes community-based education programs tailored for populations with lower education or digital literacy, and design optimization of the transmission interface for elderly users. Integrating digital emergency tool training into chronic disease management programs may foster familiarity before acute events occur.

For future research: Multicenter, large-scale prospective studies are needed to evaluate associations with clinical endpoints. Further investigation should also focus on identifying and overcoming barriers to adoption among vulnerable populations to ensure that technological advancements reduce rather than exacerbate existing health disparities^26^

In conclusion, this study demonstrates that WeChat-based prehospital data transmission represents a promising pragmatic approach, the implementation of which was associated with real-time information sharing, streamlined hospital pathways, and shorter critical time intervals in STEMI care. This experience in the Chinese context suggests that widely adopted social platforms may be utilized for healthcare improvement. Realizing its full potential will require deliberate efforts to integrate it into clinical workflows, address underlying health literacy gaps, and rigorously evaluate its long-term impact on patient outcomes across diverse populations (Fig. 4).

**Fig. 4:**
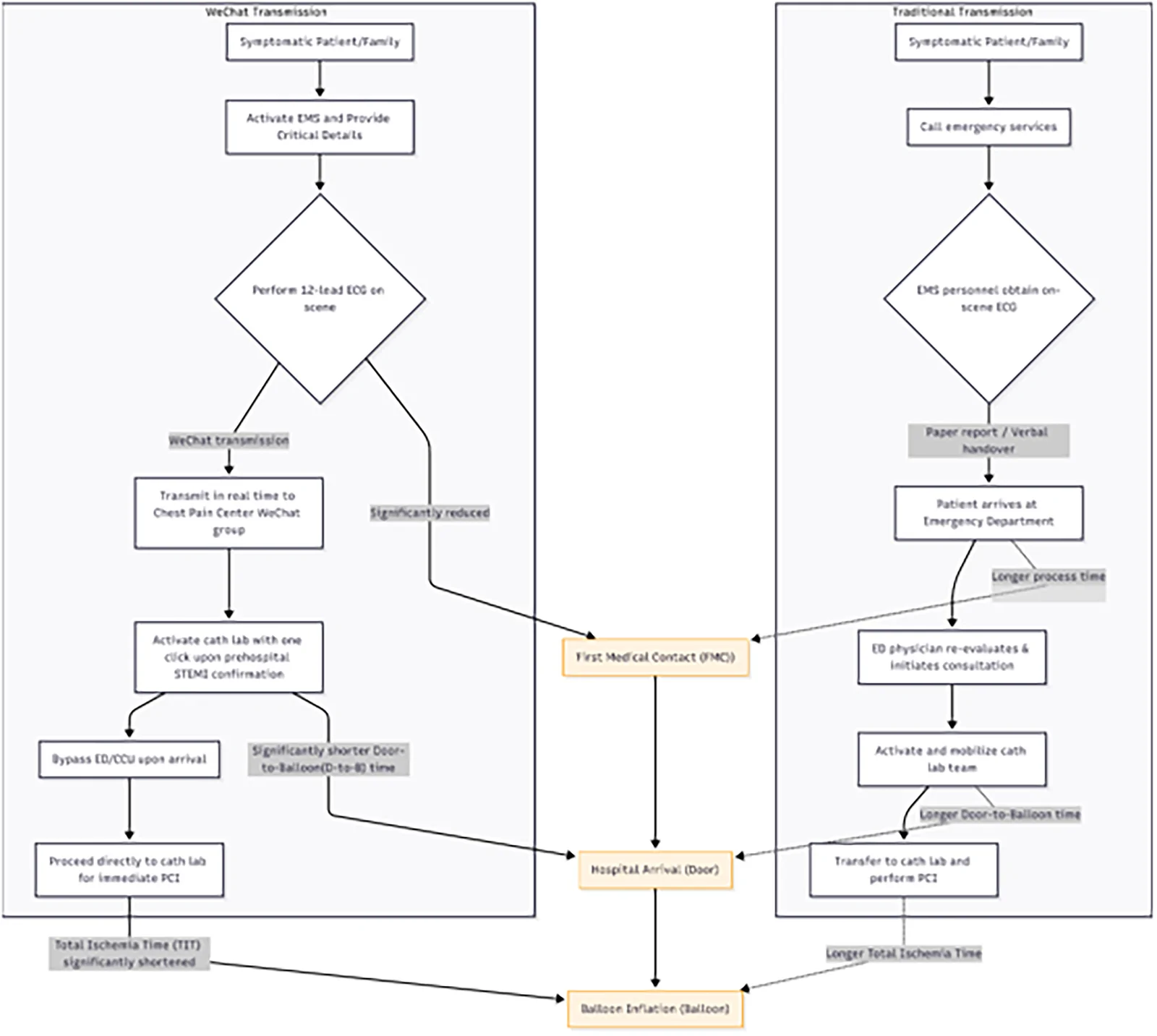
Comparison between the WeChat-based transmission pathway and the traditional pathway.

## Ethics committee information

Hebei General Hospital Ethics Committee.

## Ethics approval number

NO.2025-LW-0283(The ethical review documentation pertains to an amendment).

## Statement

This study strictly adhered to the STROBE guidelines, and all data are available within the article.

## Authors’ contributions

Shangchen Li: Conceptualization; methodology; investigation; data curation; formal analysis; writing original draft.

Haibo Wu: Investigation; Data collection; Visualization.

Ying Wang: Patient enrollment; Clinical data sorting.

Yanqing Wang: Case follow-up; Data verification.

Yuyang Xiao: Statistical analysis assistance.

Dan Yang: Laboratory index detection; Resources support.

Dongxia Liu: Conceptualization; supervision; funding acquisition; writing review & editing; project administration.

All authors have read and approved the final version of the manuscript.

## Data availability

The datasets generated and/or analyzed during the current study are available from the corresponding author upon reasonable request.

## Conflicts of interest

The authors declare no conflicts of interest.
